# A289 EXPLORING THE LIVED EXPERIENCES OF ADULT USING HOME ENTERAL NUTRITION AND THEIR CAREGIVERS: A QUALITATIVE SYSTEMATIC REVIEW

**DOI:** 10.1093/jcag/gwac036.289

**Published:** 2023-03-07

**Authors:** R Sandhu, T Elliott, J Engbers, T Edwards, W Hussain, C Johnson, T Pawliw, R Kassam

**Affiliations:** 1 Dietitian Services; 2 Fraser Health Authority; 3 Frase, Surrey; 4 Vancouver Coastal Health Authority; 5 University of British Columbia, Vancouver, Canada

## Abstract

**Background:**

Home enteral nutrition allows individuals to receive food and water through a tube at home when they are unable to meet their needs by mouth. To date, systematic literature reviews to date have focused on specific insertion methods, coordinated community care models, and prophylactic vs. reactive use. Few qualitative systematic reviews exist and those that do have only explored the lived experiences of sub-populations of adults using home enteral nutrition, such as those with head and neck cancers. Given healthcare services often serve adult home enteral nutrition populations with various underlying conditions, a synthesis describing the lived experiences of all adult using home enteral nutrition and caregivers is needed.

**Purpose:**

To conduct a meta-aggregation systematic review to evaluate lived experiences of home enteral nutrition in adult users and their caregivers.

**Method:**

A systematic search of Medline, PsychINFO, EmBase and CINAHL was conducted September 2021. Only studies with a full-text and published in English were included. Study quality was assessed using the Johanna Briggs Institute meta-aggregation methodology. Details of the study design, participants, and experiences were recorded, and themes across all studies collatedCollated themes were reviewed with a team of healthcare providers, home enteral nutrition users and a caregiver to ensure trustworthiness.

**Result(s):**

A total of 38 studies representing 702 participants were included. Using the meta-aggregative approach, three different synthesized findings were identified: (1) positive experiences, (2) negative experiences, and (3) facilitators and coping mechanisms. While, overall, the findings reported more negative experiences than positive experiences, users and caregivers who had developed coping mechanisms or had access to supportive services tended to share more positive experiences with home enteral nutrition. In contrast, those with few to no supports or coping mechanisms reported greater dissatisfaction and negative experiences.

**Image:**

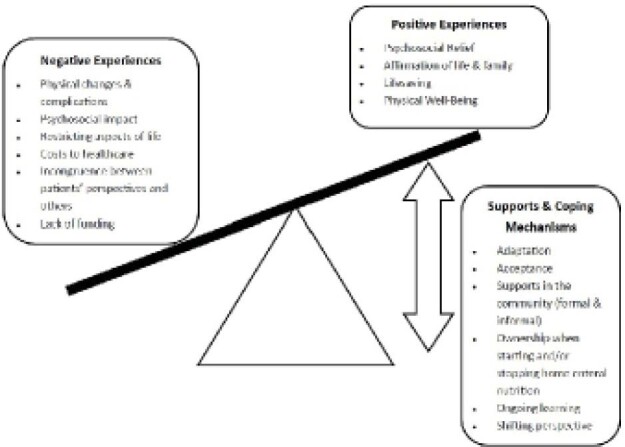

**Conclusion(s):**

Home enteral nutrition impacts the lived experience of users and their caregivers. While more negative than positive experiences were reported by adults and their caregivers using home enteral nutrition, overall, they still view home enteral nutrition as a positive intervention if they are adequate supported or developed adequate coping mechanisms. It is imperative that healthcare providers working with adults using home enteral nutrition explore and understand their patients’ and caregivers’ needs, in order to ensure community programs are designed to provide the necessary supports to maximize users experiences.

**Please acknowledge all funding agencies by checking the applicable boxes below:**

None

**Disclosure of Interest:**

None Declared

PANCREATIC DISORDERS

